# RoundMi: A quantitative method to analyze mitochondrial morphology in mitotic cells

**DOI:** 10.1002/2211-5463.70257

**Published:** 2026-05-01

**Authors:** Elmira Parvindokht Bararpour, Christine Volmert, Valentina Piano

**Affiliations:** ^1^ Institute of Human Genetics, University of Cologne, Faculty of Medicine and University Hospital Cologne Germany; ^2^ Center for Molecular Medicine (CMMC) University of Cologne Germany

**Keywords:** image‐based tool for organelle analysis, mitochondria, mitochondrial morphology analysis, mitosis

## Abstract

Mitochondrial morphology is a key readout of cellular physiology and pathophysiology, yet its quantitative analysis in mitotic cells remains technically demanding due to their rounded, three‐dimensional architecture. Volumetric imaging approaches, while comprehensive, require extensive Z‐stack acquisition, high computational resources, and specialized image analysis expertise, collectively limiting throughput and accessibility. Here, we present RoundMi, a streamlined workflow for rapid, quantitative analysis of mitochondrial morphology in mitotic cells using single focal plane imaging. RoundMi integrates automated preprocessing via the Nellie plugin in Napari with downstream segmentation and quantification in MitoSkel. Focal plane selection is guided by DNA staining and mitochondrial signal to capture representative morphological features while minimizing acquisition time and phototoxicity. We validated RoundMi in mouse embryonic fibroblasts (MEFs) and HeLa cells, demonstrating robust detection of established morphological differences between wild‐type and DRP1‐deficient cells in both interphase and mitosis. Benchmarking against volumetric methods, including deconvolution and maximum intensity projection, confirmed that single‐plane analysis provides a reliable proxy for mitochondrial morphology while avoiding projection‐induced artifacts and substantially reducing computational demand. RoundMi is applicable across multiple cell types and compatible with live‐cell imaging, offering a versatile, high‐throughput solution for mitochondrial morphology analysis in dividing cells.

## Graphical abstract

RoundMi is a workflow for rapid analysis of mitochondrial morphology in mitotic cells. By combining adaptive preprocessing with automated segmentation and quantification, it enables accurate measurements from single focal plane images, reducing acquisition time and computational demands while remaining compatible with high‐throughput fixed and live‐cell imaging.

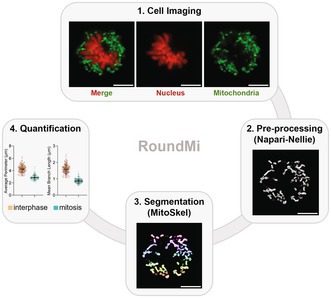

**Abbreviations**
ATP: adenosine triphosphate
Ctrl: control
DRP1: dynamin‐related protein 1
KO: knock‐out
MEF: mouse embryonic fibroblasts

Mitochondria are double‐membrane organelles that fuel cellular processes by producing ATP and act as signaling hubs to direct cellular metabolism, stress responses, immunity, and cell fate [1]. The function of mitochondria is tightly linked to their morphology: Changes in mitochondrial shape and dynamics can be both a cause and a consequence of their response to shifting cellular demands or conditions [2]. Alterations in mitochondrial number, for instance, can indicate changes in biogenesis [3], or turnover [4], while variations in size, elongation, and interconnectivity often reflect differences in metabolic states [5] or cellular stress conditions [6]. Although many different tools are available for the (semi‐)automatic quantification of the morphological features of the mitochondrial network in live and fixed cells [7, 8, 9, 10, 11, 12, 13, 14, 15, 16, 17, 18, 19], selecting the most suitable method for a specific cell type and cell state can still be challenging. Most of these tools rely on a preprocessing step that converts images to binary format to enable organelle identification and measurement. However, under certain conditions, some methods may fail to achieve a reliable segmentation, leading to inaccurate or inconsistent results [20].

A further layer of complexity arises from the fact that basal mitochondrial morphology and dynamics differ significantly among cell types and cell cycle stages [21]. In interphase cells, mitochondria exist as a heterogeneous population of interconnected tubular networks, composed of branched and singular rods that continuously fuse and divide. However, when cells round up and prepare to divide, mitochondria fragment and distribute in the cytosol to ensure their even segregation [22, 23]. These morphological changes depend on the ability of mitochondria to undergo fusion and fission events driven by the synergistic activity of dynamin‐family GTPases, such as mitofusins and dynamin‐related protein 1 (DRP1), as well as cytoskeletal proteins [2, 24]. Despite decades of research elucidating the molecular machinery of fission and fusion and their upstream regulators, the downstream integration of signaling inputs controlling these processes in different cellular contexts remains incompletely understood [25, 26]. The regulation of mitochondrial dynamics during cell division is an area of growing interest. Recent studies have demonstrated that mitochondrial distribution and morphology during mitosis are not random processes but are tightly regulated to ensure faithful segregation of mitochondrial mass and function to daughter cells. Moreover, mitochondrial segregation is important for establishing asymmetry and determining cell fate. [27, 28, 29, 30, 31]. Perturbations in these processes have been linked to mitochondrial dysfunction in progeny, with potential consequences for cellular differentiation and development [32, 33, 34].

Still, robust quantitative tools to systematically assess mitochondrial morphology specifically in mitotic cells have been lacking, in part due to the unique technical challenges associated with imaging these cells. In fact, mitotic cells undergo a dramatic reorganization of their cytoskeleton [35, 36]. As a result, although they have roughly twice the volume of their interphase counterparts, they appear more compact than adherent interphase cells, and mitochondria become more crowded and heterogeneous, often displaying variable fluorescence intensity due to their distribution along the Z‐axis.

To address these challenges, we developed and validated RoundMi, a new workflow for rapid and reliable analysis of mitochondrial morphology in mitotic cells imaged in a single focal plane. RoundMi uses the freely available Napari–Nellie [37] for adaptive preprocessing of fluorescence images, and MitoSkel [38] for downstream segmentation and morphological quantification. While MitoSkel's original preprocessing pipeline was trained on images of interphase cells and performs poorly with confocal images of rounded mitotic cells, combining Napari–Nellie's adaptive pre‐processing with MitoSkel's segmentation algorithms overcame these limitations. Napari–Nellie also provides organelle segmentation after preprocessing, however, while segmentation was generally accurate for interphase cells, in rounded mitotic cells, Napari–Nellie often failed to delineate individual mitochondria.

By integrating Napari–Nellie and MitoSkel, RoundMi provides a practical 2D approximation for quantifying mitochondrial morphology in mitotic cells from confocal images, reducing reliance on 3D reconstruction for comparative analyses. This workflow addresses a key technical limitation of existing approaches, extends mitochondrial morphology analysis to mitotic cells, reduces the time of acquisition and analysis of the images, and offers an accessible platform for both basic and translational studies of mitochondrial dynamics.

## Materials and methods

### Cell culture conditions and synchronization

Mouse embryonic fibroblasts (MEF) DRP1 knock‐out (KO) and MEF control (ctrl) cells were provided by the laboratory of Ana Garcia‐Saez (Max Planck Institute, Frankfurt, Germany). HeLa DRP1‐KO cells were generated and provided by the laboratory of Thomas Langer (Max Planck Institute, Cologne, Germany). Cell lines were cultured according to standard protocols. Briefly, DLD‐1, hTERT RPE‐1, and U2OS cells were grown in DMEM/F12 (Gibco, Thermo Fisher Scientific), whereas HeLa and MEF ctrl and DRP1‐KO cells were cultured in DMEM (high glucose; Gibco, Thermo Fisher Scientific). All media were supplemented with 10% (v/v) fetal bovine serum (FBS) and 1% (v/v) penicillin/streptomycin. Cells were routinely passaged using Trypsin–EDTA (1×; Sigma‐Aldrich) and maintained at 37 °C in a humidified incubator with 5% CO_2_.

To promote adhesion of mitotic cells to the glass slide, the slides were coated with Poly‐L‐Lysine prior to cell seeding for immunofluorescence. Poly‐L‐Lysine was diluted 1 : 25 in PBS and added to the wells, then incubated for 1 h (37 ° C, 5% CO_2_). Afterward, wells were washed three times with PBS prior to seeding. For immunofluorescence, 50 000 cells were seeded in each well of a 4‐well chamber slide (Millicell, Millipore), and for live‐cell imaging, cells were seeded in a 4‐well glass‐bottom chamber slide (Ibidi GmbH). Cells were synchronized 24 h after seeding by adding RO‐3306 (Selleck) to a final concentration of 9 μm. After 16–18 h, cells were washed four times with PBS and incubated in fresh medium for 30 min. MG132 (Selleck) was then added to a final concentration of 10 μm, and cells were incubated for 30–60 min at 37 °C prior to fixation or live‐cell imaging.

### Immunofluorescence and live‐cell imaging of cultured cells

Immunofluorescence was performed after synchronization with RO‐3306 and MG132, followed by fixation with 4% paraformaldehyde (PFA) for 10–15 min at room temperature (RT). Cells were washed 3 times with PBS and permeabilized with PBS containing 0.2% Tween‐20 for 5 min at RT. Cells were then blocked with blocking solution (PBS, 0.2% Tween‐20, 2% BSA, 1% FBS) for 1 h at RT and incubated overnight at 4 °C with anti‐TOMM20 antibody (Abcam, AB186735) diluted 1 : 1000 in blocking solution. The following day, cells were washed three times with PBS and incubated with the secondary antibody (Alexa Fluor™ 568 or 488 donkey anti‐rabbit IgG, Invitrogen) diluted 1 : 250 in blocking solution for 3 h at 4 °C. Cells were then washed twice with PBS, stained with NucBlue (Invitrogen) for 5 min, and washed again with PBS. Finally, samples were rinsed with water and mounted using ProLong™ Gold Antifade Reagent (Invitrogen). Slides were dried for 1 h at RT before imaging and stored at 4 °C if required.

Live‐cell imaging was performed after synchronization treatment with RO‐3306. Cells were then stained with 100 nM MitoTracker™ Green (#M7514; Thermo Fisher Scientific) and SiR‐DNA (Tebubio) for 30 min at 37 °C. Following staining, cells were treated with MG132 for 30 min. The medium was exchanged for phenol red–free DMEM imaging medium (Gibco, Thermo Fisher Scientific) before live‐cell imaging.

### Confocal fluorescence microscopy

Cells were imaged using a Leica STELLARIS 5 laser‐scanning confocal microscope (Leica Microsystems) mounted on an inverted DMi8 stand and equipped with a 63×/1.30 glycerol‐immersion objective (HC PL APO). Alexa Fluor™ 568 and Alexa Fluor™ 488 donkey anti‐rabbit IgG were excited at 579 nm and 488 nm, respectively, and DAPI at 405 nm. Emission signals were detected using four Power HyD S hybrid detectors (Leica Microsystems) and a transmitted light photomultiplier tube (T‐PMT). Images were acquired with a pixel size of 43.83 nm at a zoom factor of 2.25 or 65.70 nm at zoom factor of 1.5, a pixel dwell time of 226 ns, line averaging of 4 or 7, and a pinhole diameter set to 1 Airy unit. For live‐cell imaging, we used the same settings above under temperature‐controlled conditions at 37 °C with 5% CO_2_.

Z‐stacks were collected from the middle of the cell with a Z‐step size of 0.20 μm over five steps (total Z‐stack thickness: 0.80 μm) for deconvolution. Z‐stacks for maximum projection were acquired with a Z‐step size of 0.4 μm from the bottom to the top of the cell, with 10–20 steps for interphase cells and 30–50 steps for mitotic cells. The raw images acquired from the confocal microscope are saved in “.lif” file format, which contains the complete dataset along with all associated metadata.

### Image analysis using deconvolution

Image deconvolution was performed using the Huygens Professional software (Scientific Volume Imaging B.V., The Netherlands). Confocal image stacks were imported in ‘.lif’ format, and the Deconvolution Wizard was initiated via the ‘Enter Wizard’ option. A theoretical point spread function (PSF) was automatically generated by the software. No cropping was applied to the datasets. Deconvolution was performed on Channel 1, corresponding to the TOMM20‐labeled mitochondria. The initial value strategy was set to Standard, and the deconvolution algorithm was Classic Maximum Likelihood Estimation (MLE). The signal‐to‐noise ratio (SNR) was set to 7 for HeLa ctrl and DRP1‐KO images, and to 14 for MEF ctrl and DRP1‐KO images, in line with the software's suggested values. Background estimation for channel 1 was performed automatically by the software (estimation mode: Lowest). In the Deconvolution Setup, the quality threshold was set to 0.1, maximum iterations were set to 30, and image acuity was adjusted to −50 (smoother setting). Following deconvolution, images were saved in OME‐TIFF format for further analysis. The optimal focal plane for segmentation was selected from the deconvolved Z‐stack. Individual cells were isolated by removing the background, and brightness and contrast (B&C) parameters were optimized for each image individually in imagej/Fiji. The processed images were subsequently converted to RGB format and further analyzed for segmentation and mitochondrial quantification using MitoSkel software, as described in detail in the RoundMi workflow section.

### Image analysis using max intensity projection

Z‐stacks were acquired as described above and opened in imagej/Fiji. Single cells were selected by deleting the background in all planes. Maximum intensity projections were generated using the Z Project function. The images were subsequently preprocessed using Napari–Nellie and segmented using MitoSkel, as described in detail in the following section.

### Image analysis with RoundMi workflow

Software required:

imagej/Fiji (https://fiji.sc/).

Napari (https://napari.org/stable/).

Nellie (https://github.com/aelefebv/nellie).

MitoSkel (https://github.com/SoumayaZgh1/MitoSkel?tab=readme‐ov‐file).

**Fig. 1:**
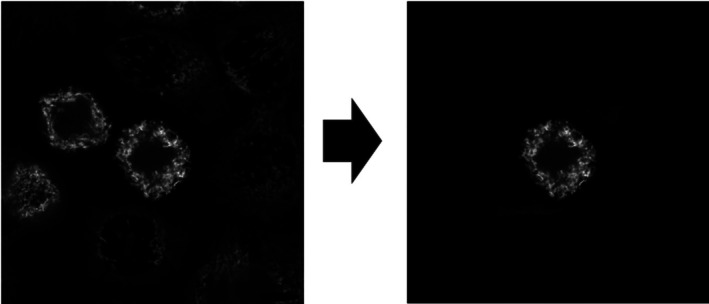
Single‐cell selection. Raw images were opened in imagej/Fiji, and single cells were selected by deleting the background. Brightness and contrast (B&C) parameters were optimized individually for each image.

**Fig. 2:**
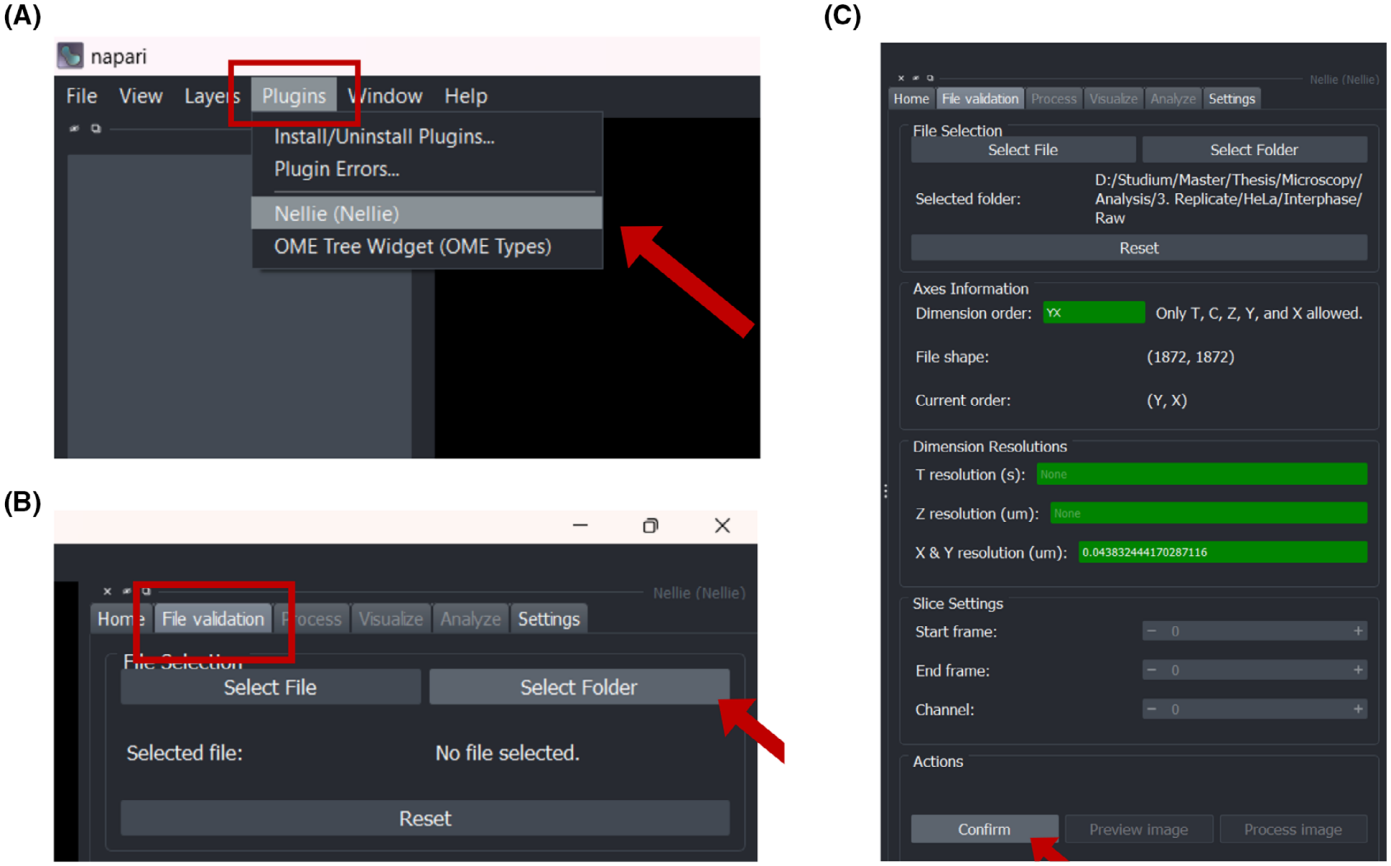
Starting the Nellie plugin and loading images. (A) Napari was opened, and the Nellie plugin (version 0.4.1) was launched. (B) Images were loaded by selecting the folder through the ‘File validation’ option. (C) After selecting the folder, ‘Confirm’ was clicked to proceed.

**Fig. 3:**
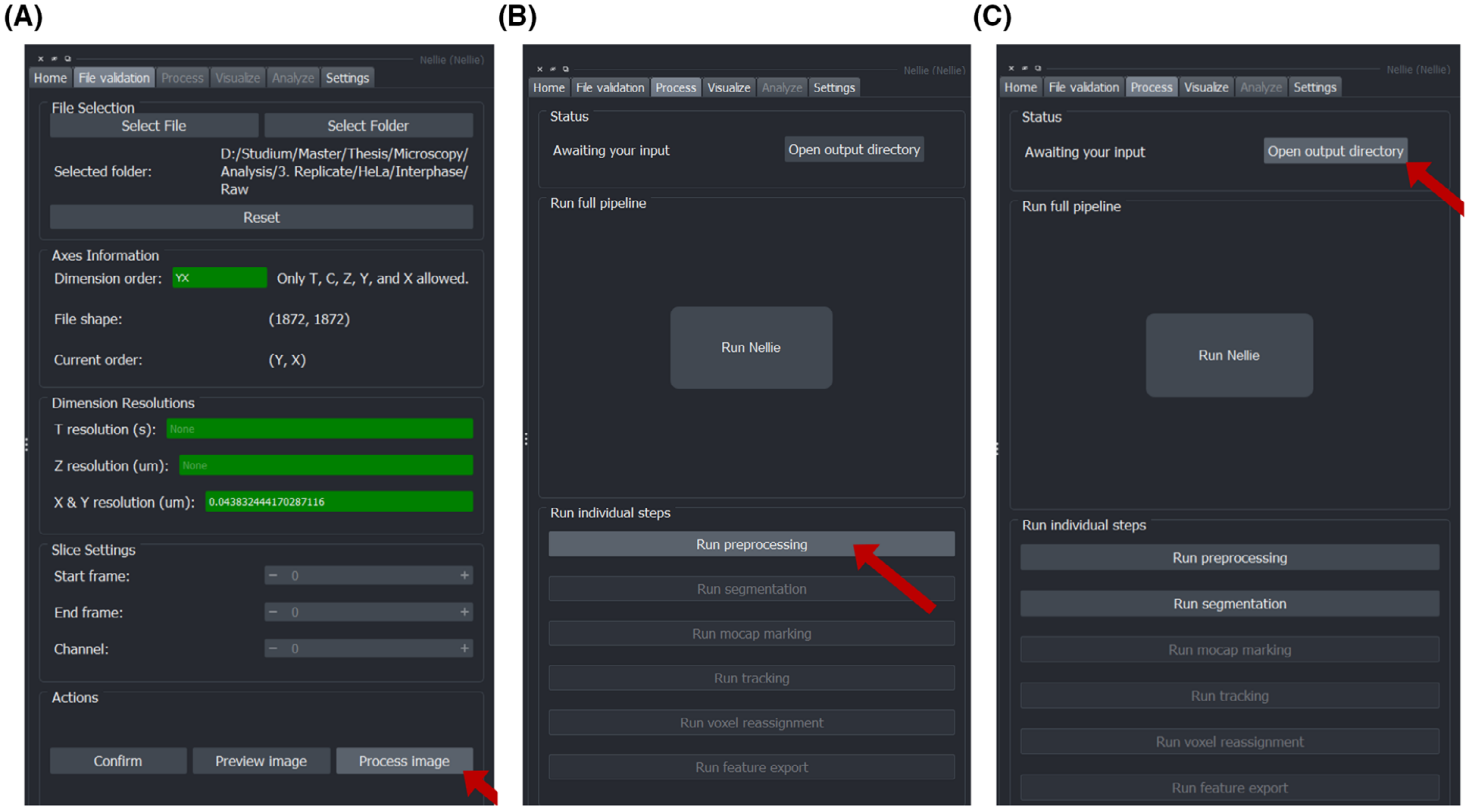
Image preprocessing with Nellie. (A) ‘Process image’ was selected. (B) ‘Run pre‐processing’ was selected. (C) ‘Open output directory’ was selected to access the folder containing the preprocessed image files (‘nellie_output’), which also includes the raw images. Preprocessed images were then transferred to a separate folder for subsequent analysis with MitoSkel.

Image analysis using the RoundMi workflow involved preprocessing and mitochondrial segmentation steps. First, raw images were opened in imagej/Fiji, and single cells were selected by removing the background. Brightness and contrast were adjusted individually for each image to improve visualization quality (Fig. 1). Next, images were preprocessed using Napari with the Nellie plugin (version 0.4.1). The plugin was launched within Napari, and images were loaded by selecting the corresponding folder via the ‘File validation’ option, followed by confirmation to proceed (Fig. 2). Image preprocessing was then performed by selecting ‘Process image’ followed by ‘Run pre‐processing’. The resulting files were accessed through the output directory (‘nellie_output’), which contains both raw and preprocessed images. Preprocessed images were then transferred to a separate folder for subsequent analysis (Fig. 3).

**Fig. 4:**
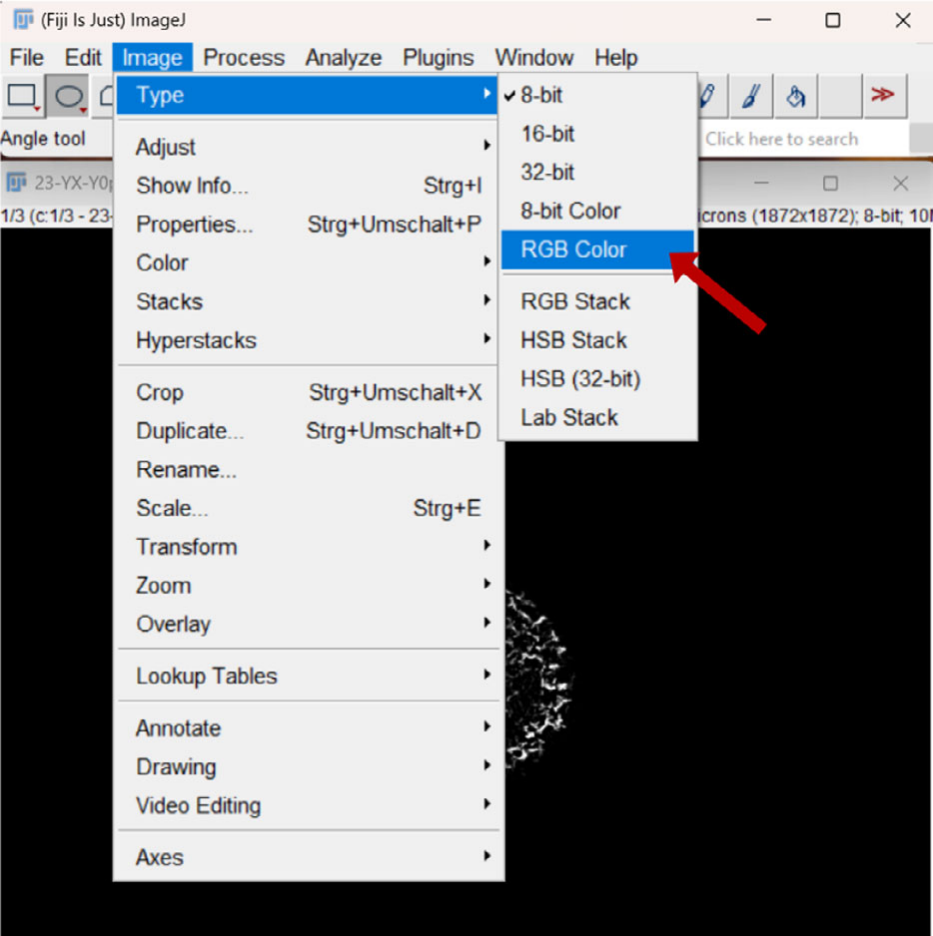
Conversion of preprocessed images for MitoSkel. Preprocessed images were opened in imagej/Fiji, converted to ‘RGB color’ format, and saved in the same folder.

**Fig. 5:**
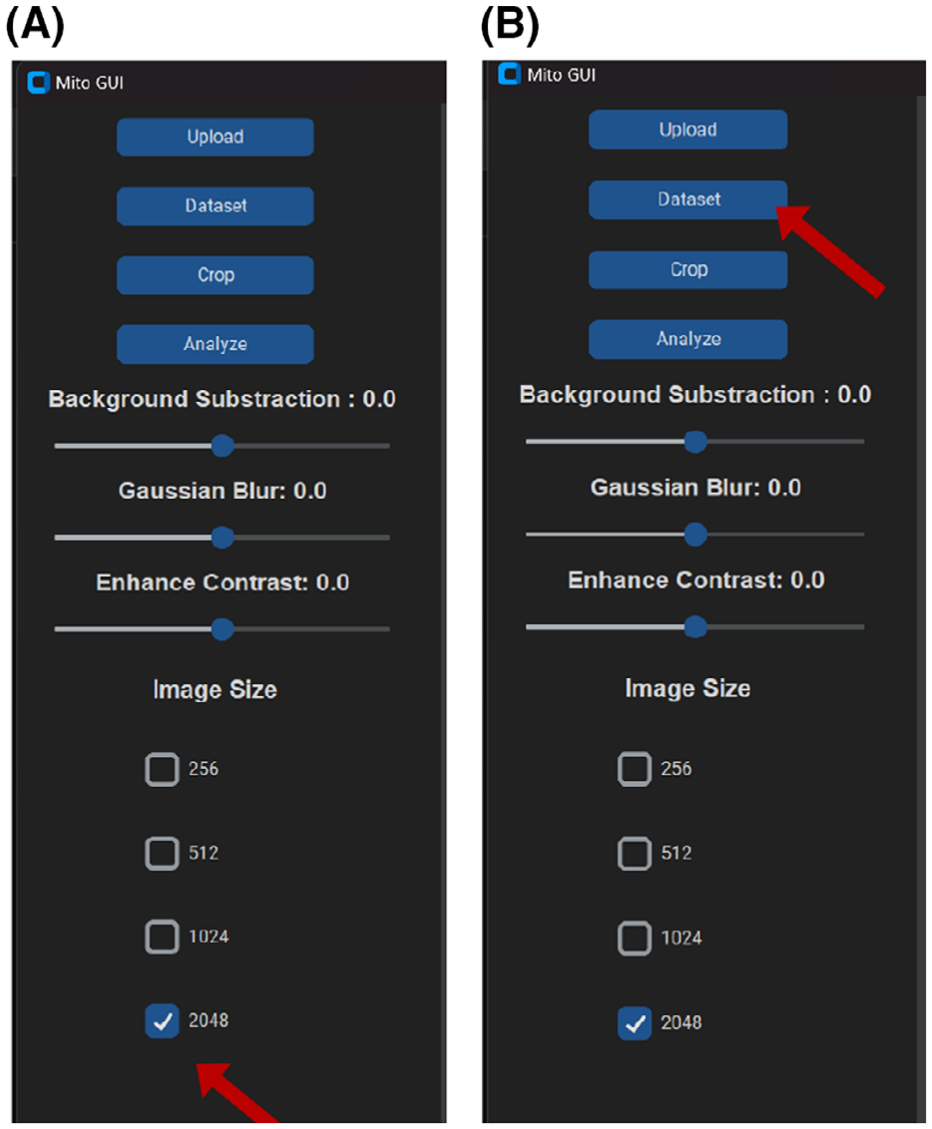
Segmentation and mitochondrial analysis with MitoSkel. (A) MitoSkel was opened, and the image size was set to 2048. (B) ‘Dataset’ was selected to load the folder containing images pre‐processed with Napari‐Nellie and converted to RGB color. Segmented images and the Excel file ‘all_contour_data’, containing the mitochondrial morphology analysis, were saved in a new folder named ‘processed_images’.

For compatibility with downstream analysis, preprocessed images were converted to RGB color format (Fig. 4). Finally, mitochondrial segmentation and analysis were carried out using MitoSkel. The image size was set to 2048, and the dataset containing the processed images was loaded. The segmented images and the Excel file ‘all_contour_data’, containing mitochondrial morphology measurements, were saved in a new folder named ‘processed_images’ (Fig. 5).

### Statistical analysis of the morphological parameters of mitochondria

The output of MitoSkel provides the analysis of mitochondria in pixels. Given the pixel size (43.83 or 65.70 nm, depending on the acquisition settings) and the confocal lateral resolution (~200 nm), detections smaller than 2 pixels are considered below the resolution limit. Accordingly, detections smaller than 2 pixels were excluded from the analysis. For each analyzed cell, the mean values of all parameters (total branch length, perimeter, area, and circularity) were calculated and converted from pixel to μm using *R*. The final dataset, comprising average parameter values for each cell across all replicates, was analyzed and visualized using graphpad Prism. Pairwise comparisons indicated in the plots were assessed using two‐way ANOVA statistical analysis.

## Results

Our aim was to develop an accessible workflow to quantify mitochondrial morphology in mitotic cells that balances accuracy with practical considerations for high‐throughput analysis. While full 3D reconstruction provides complete spatial information, it requires extensive Z‐stack acquisition, substantial computational power, and advanced image‐analysis expertise, factors that can limit throughput and accessibility.

We chose to acquire and analyze single focal planes (specifically, the middle plane) for several reasons: (1) it dramatically reduces acquisition time, enabling analysis of larger sample sizes; (2) it minimizes photobleaching and phototoxicity, critical for live‐cell imaging applications; (3) it requires less computational resources, making the approach more accessible; and (4) it enables the analysis of the mitochondria within the entire focal plane and not only a region of interest.

**Fig. 6:**
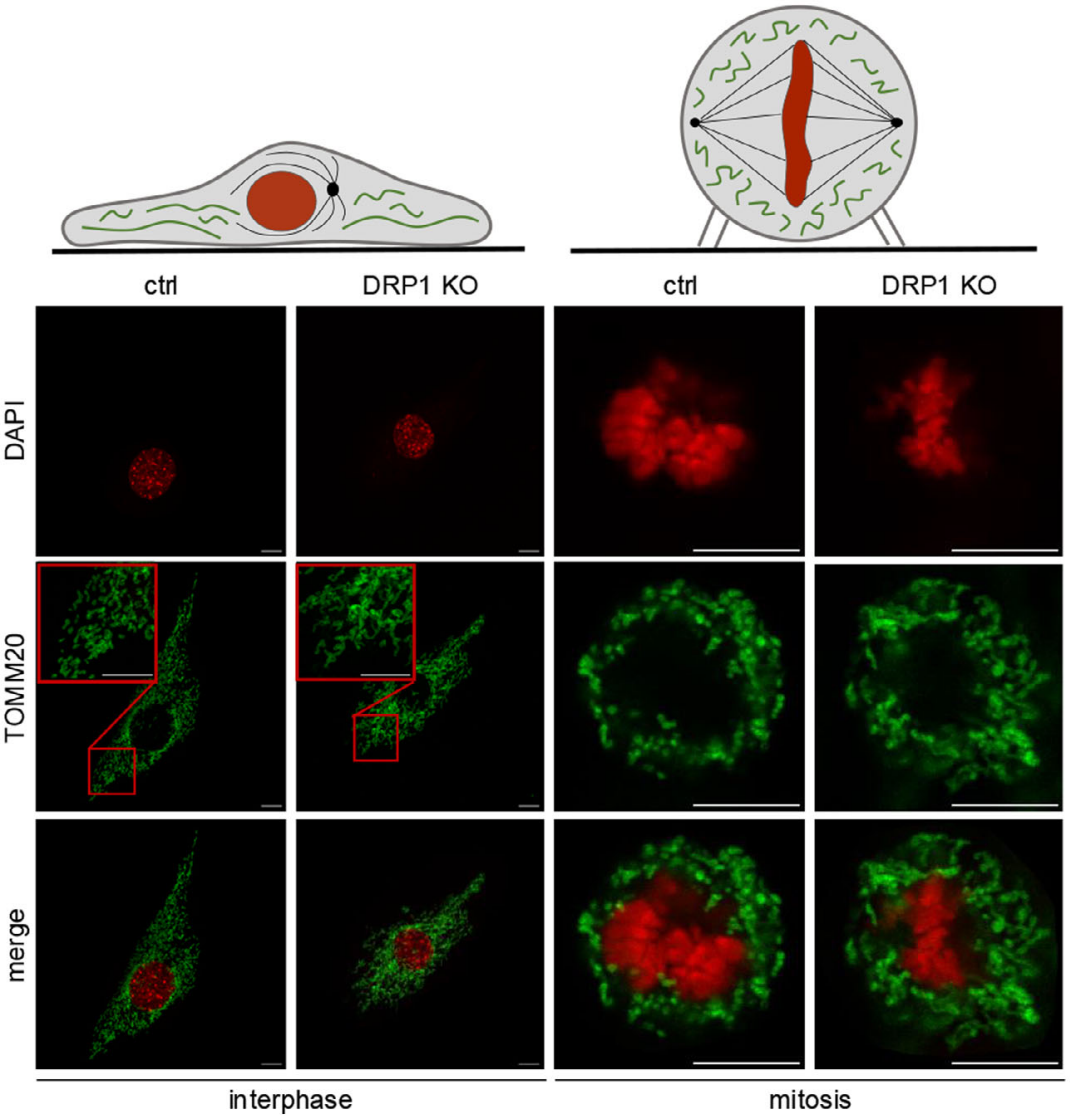
Cellular morphology during interphase and mitosis. Representative confocal images of ctrl and DRP1‐KO MEF cells in interphase and mitosis, highlighting differences in cell shape, nuclear/chromosome morphology, and mitochondrial network topology. Cells were stained for DNA (DAPI, red) and mitochondria (TOMM20, green). Schematics shown above each cell cycle stage illustrate the corresponding cell architecture and the arrangement of the nucleus and mitochondria. Images of mitotic cells were cropped in imagej/Fiji to provide a slightly zoomed‐in view. All scale bars: 10 μm.

To determine whether the RoundMi workflow (Figs 1, 2, 3, 4, 5) can capture biologically relevant differences in mitochondrial morphology, we acquired confocal images of mouse embryonic fibroblasts (MEFs), including control cells and cells depleted of the mitochondrial fission factor DRP1, which is essential for mitochondrial fragmentation (Fig. 6). As reported previously, DRP1 knock‐out (KO) cells exhibit more elongated mitochondria compared to controls in both interphase and mitotic cells [39, 40, 41, 42, 43, 44]. To further validate the method technically, we compared single focal plane analysis with two alternative volumetric acquisition strategies. This comparison allowed us to assess whether single‐plane imaging provides a reliable proxy for mitochondrial morphology analysis.

To identify the middle focal plane of mitotic cells, DNA staining was used to detect mitotic cells and provide a structural reference, and the plane corresponding to the largest cell circumference was determined based on the mitochondrial signal (Fig. 6).

**Fig. 7:**
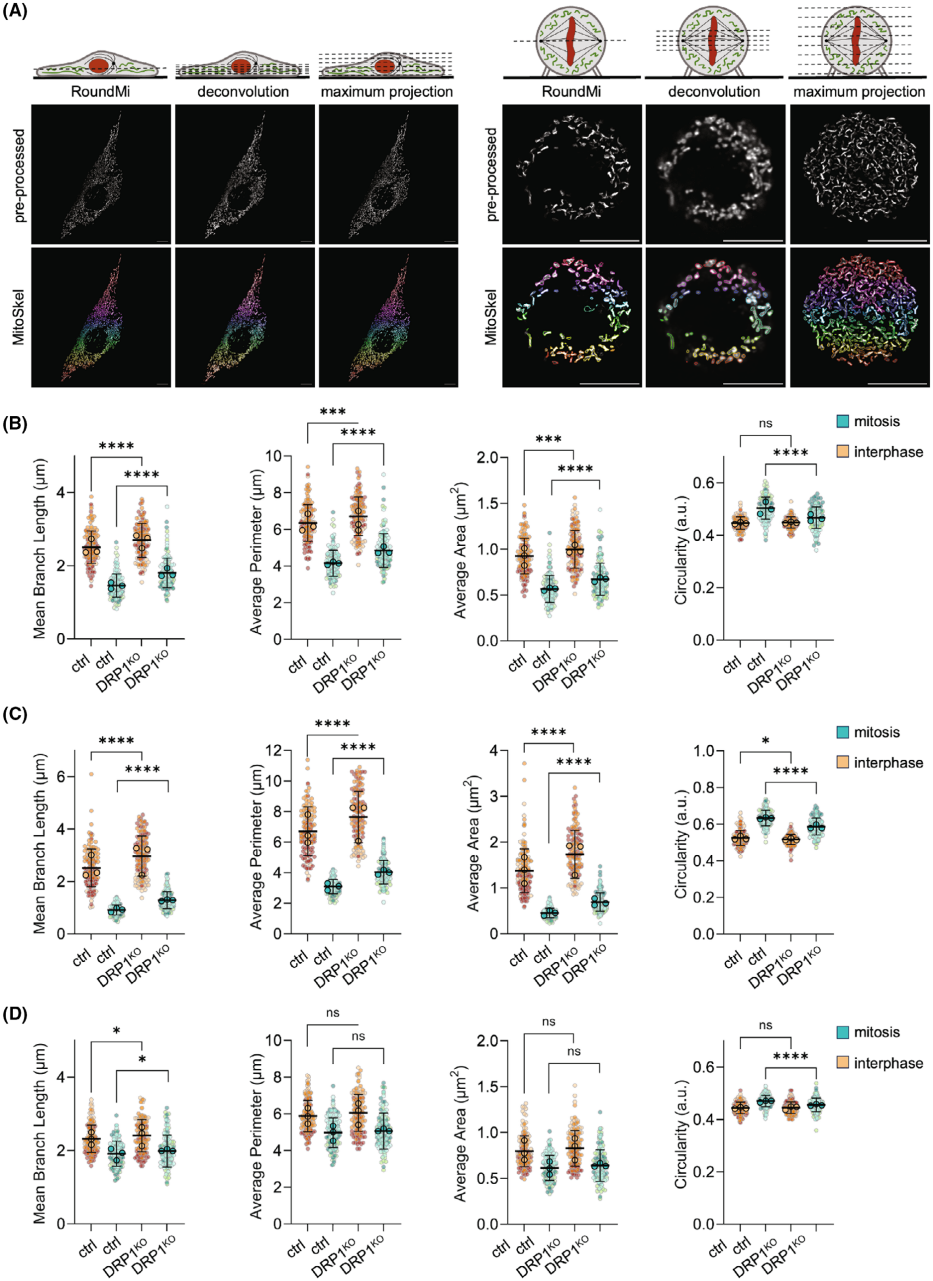
RoundMi enables robust mitochondrial morphology analysis in MEF cells compared to conventional preprocessing approaches. (A) Representative confocal images of fixed MEF control (ctrl) during interphase and mitosis, processed using three preprocessing workflows: RoundMi (Napari–Nellie), deconvolution, and maximum intensity projection (Z‐projection in imagej/Fiji and Napari–Nellie). Corresponding mitochondrial segmentations generated using MitoSkel are shown. Schematics above each workflow illustrate the image acquisition strategy in mitotic cells: a single focal plane for RoundMi, five central Z‐planes for deconvolution, and full Z‐stacks from bottom to top for maximum intensity projection. TOMM20 is used to visualize mitochondria. Scale bars: 10 μm. (B–D). Quantification of mitochondrial morphology parameters in ctrl and DRP1‐KO MEF cells during interphase and mitosis following different preprocessing workflows: (B) RoundMi, (C) deconvolution, and (D) Napari‐Nellie after maximum intensity projection. Parameters include mean total branch length, average perimeter, average area, and circularity. Data represents *n* = 3 independent experiments, with at least 50 cells analyzed per replicate (~150 cells total) per condition. Transparent circles represent individual cells, with different colors indicating independent replicates. Filled circles indicate the mean of each replicate, the black line indicates the mean of all cells and error bars represent standard deviation. Color coding for interphase and mitosis is indicated in the figure. Pairwise comparisons between conditions were assessed using two‐way ANOVA statistical analysis. *P < 0.05; **P < 0.01; ***P < 0.001; ****P < 0.0001; ns, not significant (*P* > 0.05).

We then selected a single cell for field of view (Fig. 1) and uploaded it into Napari–Nellie for preprocessing (Figs 2, 3, 7A). Mitochondrial fluorescence signal quality is strongly influenced by experimental factors such as microscope system, laser power, and dye choice. These variables can complicate organelle discrimination due to variability in image brightness and contrast. Napari–Nellie applies an advanced implementation of the *Frangi* filter [45] that automatically adapts to structures within the organelle size range, improving preprocessing robustness across diverse imaging conditions. Comparable enhancement of image quality can be achieved with deconvolution algorithms [46], which computationally reduce out‐of‐focus blur and improve definition of subcellular features. However, optimal deconvolution may require parameter optimization and user expertise, and is dependent on the particular software employed.

We compared the preprocessing of Napari–Nellie of a single focal plane to deconvolution. Because accurate deconvolution requires depth information, the original Z‐stack was processed in Huygens Professional as described in the Methods section. Deconvolution algorithms operate in three dimensions to reverse optical blur, and therefore rely on Z‐stack data to restore sharpness throughout the volume. From the deconvolved Z‐stack, we again selected the same central focal plane used for Napari–Nellie preprocessing (Fig. 7A). In addition, we compared single‐plane analysis to maximum intensity projections (see Methods) of the full Z‐stack (from the bottom to the top of the cells), which were preprocessed using Napari–Nellie (Fig. 7A). The preprocessed images were then converted in imagej/Fiji to RGB type which is compatible with the MitoSkel software (Fig. 4).

After segmentation with MitoSkel (Figs 5 and 7A), we obtained datasets containing pixel‐based morphometric measurements of the segmented mitochondria across all volumetric acquisitions and preprocessing methods (Napari–Nellie applied to a single focal plane, decovolution with partial Z‐stack and Napari–Nellie applied to maximum intensity projection). From the MitoSkel output, we calculated the average of total branch length, perimeter, area, and circularity of all mitochondria per each single cell. These pixel‐based values were converted to micrometers based on the original image pixel size.

Comparison of mitochondrial morphology in cells analyzed with RoundMi and deconvolution‐MitoSkel showed that mitochondria are generally more elongated in interphase cells compared to mitotic cells (Fig. 7B,C). Importantly, both methods reliably detected significant differences across all morphological parameters between control and DRP1‐KO MEFs (Fig. 7B,C), consistent with the expected hyperfused mitochondrial phenotype in DRP1‐deficient cells [44]. In contrast, maximum intensity projection introduced systematic distortions in mitochondrial morphology segmentation (Fig. 7A), likely due to the compression of three‐dimensional structures into a two‐dimensional plane, which can artificially merge overlapping mitochondrial networks resulting in inaccurate measurements (Fig. 7D).

**Fig. 8:**
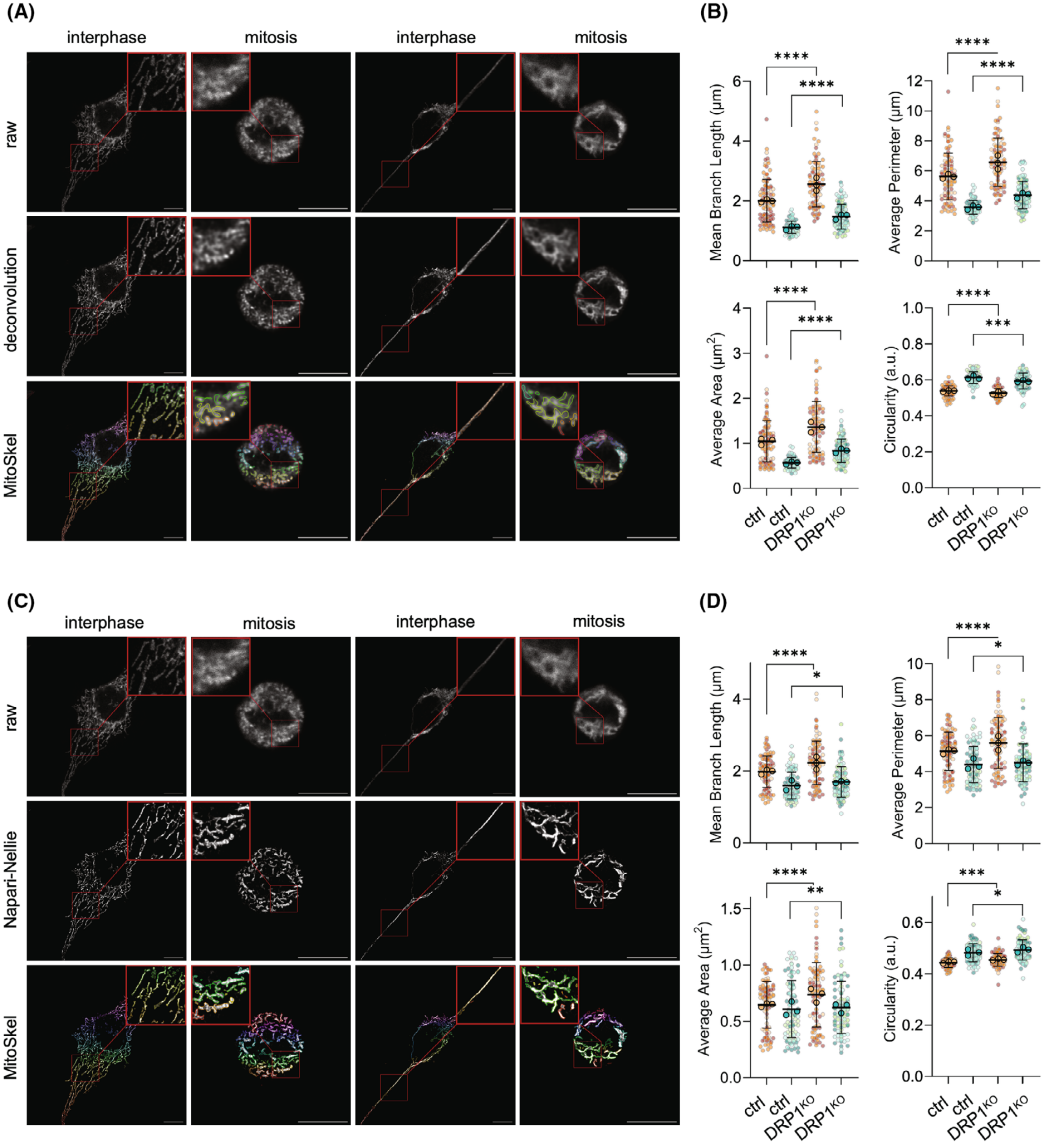
Validation of RoundMi for mitochondrial morphology analysis in HeLa cells. (A) Representative confocal images of fixed HeLa ctrl and DRP1‐KO cells during interphase and mitosis. Raw and deconvolved images are shown together with the corresponding mitochondrial segmentations generated using MitoSkel. TOMM20 is used to visualize mitochondria. Scale bar: 10 μm. (B) Quantification of mitochondrial morphology parameters in ctrl and DRP1‐KO HeLa cells during interphase and mitosis following deconvolution‐based preprocessing. Parameters include mean total branch length, average perimeter, average area, and circularity. (C) Representative confocal images of fixed HeLa ctrl and DRP1‐KO cells preprocessed using the RoundMi workflow (Napari–Nellie), with corresponding MitoSkel segmentations. Scale bar: 10 μm. (D) Quantification of mitochondrial morphology parameters as in (B), following RoundMi workflow. Data represents *n* = 3 independent experiments, with at least 30 cells analyzed per replicate (~100 cells total) per condition. Transparent circles represent individual cells, with different colors indicating independent replicates. Filled circles indicate the mean of each replicate, the black line indicates the mean of all cells and error bars represent standard deviation. Color coding for interphase and mitosis is indicated in the figure. Pairwise comparisons between conditions were assessed using two‐way ANOVA statistical analysis. *P < 0.05; **P < 0.01; ***P < 0.001; ****P < 0.0001; ns, not significant (*P* > 0.05).

These results demonstrate that RoundMi captures biologically meaningful differences in mitochondrial morphology associated with both cell cycle stage and genetic perturbations, consistent with prior reports [41, 47, 48, 49, 50]. Similarly, cells preprocessed using deconvolution exhibited mitochondrial morphology patterns comparable to those obtained with RoundMi (Fig. 7B,C). These trends were also recapitulated in HeLa cells, that we analyzed both using RoundMi and deconvolution‐MitoSkel (Fig. 8A–D), thus showing that RoundMi accurately captures mitochondrial morphological changes across different cell lines.

**Fig. 9:**
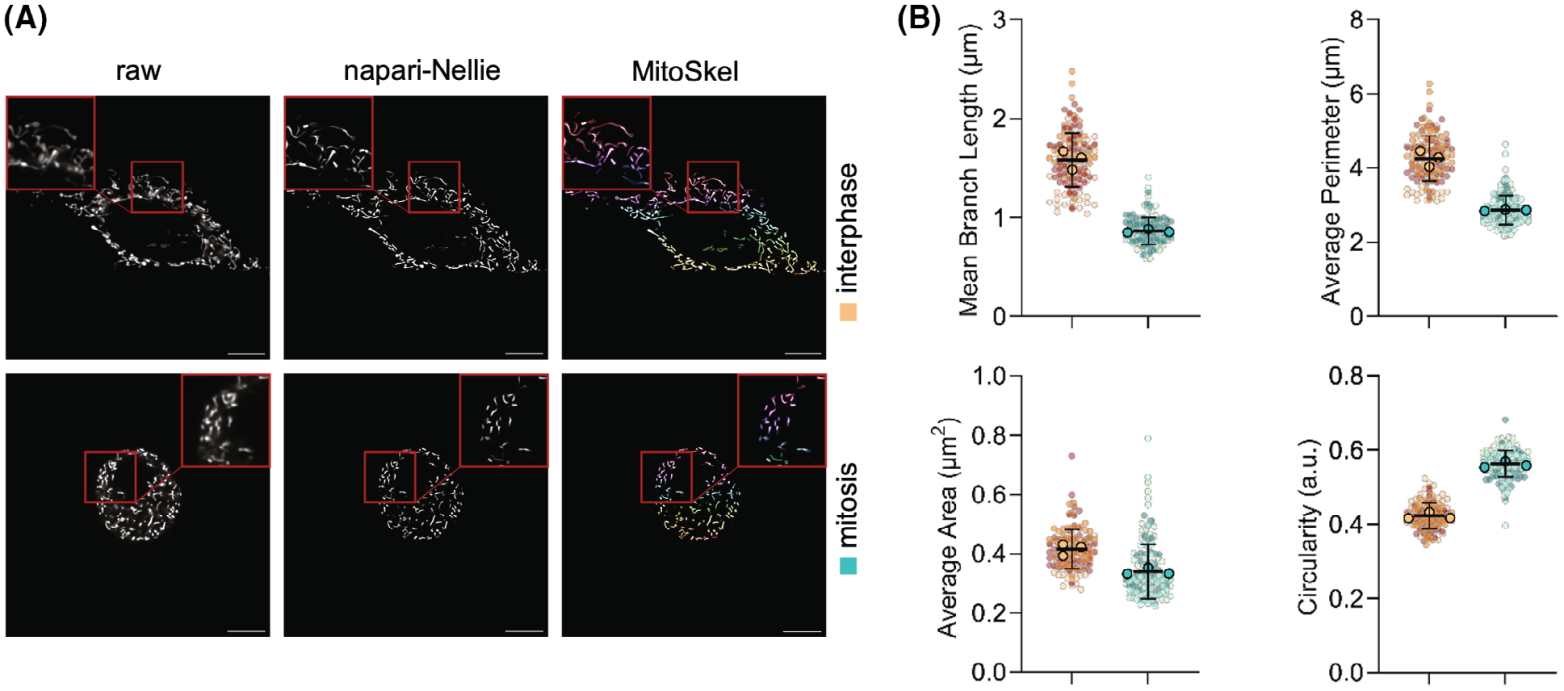
Application of RoundMi to mitochondrial morphology analysis in live‐cell imaging. (A) Representative confocal images of live MEF ctrl cells during interphase and mitosis. Raw images, images pre‐processed using Napari–Nellie, and the corresponding mitochondrial segmentations generated using MitoSkel are shown. Images were cropped in imagej/Fiji to provide a zoomed‐in view. Mitochondria were labeled with MitoTracker Green. Scale bar: 5 μm. (B) Quantification of mitochondrial morphology parameters in live MEF ctrl cells during interphase and mitosis following the RoundMi workflow, including mean total branch length, average perimeter, average area, and circularity. Data represents *n* = 3 independent experiments, with at least 50 cells analyzed per replicate (~150 cells total) per condition. Transparent circles represent individual cells, with different colors indicating independent replicates. Filled circles indicate the mean of each replicate, the black line indicates the mean of all cells and error bars represent standard deviation. Color coding for interphase and mitosis is indicated in the figure.

Furthermore, we performed confocal live‐cell imaging of control MEFs in interphase and mitosis (Fig. 9A) and confirmed the suitability of RoundMi to live‐cell imaging by quantifying all mitochondrial morphological parameters (Fig. 9B).

Finally, to test the general applicability of RoundMi across commonly used cell lines, we acquired and analyzed confocal images of HeLa (Fig. S1A,B), DLD‐1 (Fig. S1C,D), hTERT‐RPE‐1 (Fig. S1E,F), and U2OS cells (Fig. S1G,H) during both interphase and mitosis. Because RoundMi requires only a single focal plane, acquisition time was markedly reduced and, despite differences in cell size, morphology, and mitochondrial density, RoundMi successfully enabled preprocessing, segmentation, and analysis across all tested cell types.

## Discussion

We present RoundMi, a new workflow for the quantitative analysis of mitochondrial morphology in mitotic cells. This approach integrates two freely available analysis tools: Napari–Nellie [37] and MitoSkel [38].

RoundMi reliably detected biologically expected differences in mitochondrial morphology between control and DRP1‐KO cells, in both interphase and mitotic populations. Given the established role of DRP1 in mitochondrial fission, its depletion leads to elongated mitochondrial networks, and this phenotype was consistently captured by our workflow. While absolute values of mitochondrial parameters may differ from those obtained using alternative approaches, RoundMi robustly detects relative differences between conditions.

A key feature of RoundMi is its ability to extract quantitative information from single focal plane images, thereby reducing the need for full 3D reconstruction. Although volumetric imaging provides a more complete representation of mitochondrial architecture, it is time‐consuming, computationally intensive, and less compatible with high‐throughput or live‐cell applications. In contrast, single‐plane acquisition enables faster imaging, reduces phototoxicity, and simplifies data analysis, making the workflow more accessible to users with varying levels of expertise. This is particularly advantageous for live‐cell imaging, where minimizing acquisition time is critical. However, this simplification also represents a limitation, as information along the Z‐axis is not fully captured and may influence the interpretation of complex mitochondrial networks.

By combining Napari–Nellie's robust preprocessing capabilities with MitoSkel's automated analysis, RoundMi enables accurate and reproducible quantification of mitochondrial morphology in the challenging context of rounded mitotic cells. This workflow extends the use of existing analysis tools to mitotic cells and provides a practical, scalable, and accessible platform for both fixed and live‐cell studies of mitochondrial dynamics in dividing cells.

## Conflict of interest

The authors declare no conflict of interest.

## Author contributions

EPB and CV were involved in methodology development, sample preparation, data analysis, draft, figure preparation, final edit, revision. VP was involved in supervision, resources, support in analysis, evaluation of the results, final edit, revision.

## Supporting information


**Fig. S1.** Application of RoundMi to multiple cell lines.

## Data Availability

All raw data supporting the method development of this study are publicly available in the repository BioStudies, accession number S‐BSST2915. The repository includes the original microscopy datasets used for the quantitative analysis described. All files are provided in formats that allow reproduction of the analyses described in this article.
