# Supplementary material for: RoundMi: A quantitative method to analyze mitochondrial morphology in mitotic cells
Source: FEBS Open Bio. 2026 May 1:10.1002/2211-5463.70257. Online ahead of print. doi: 10.1002/2211-5463.70257 (PMC13398926; doi:10.1002/2211-5463.70257)
Supplement: Supplementary file 1 — Fig. S1. Application of RoundMi to multiple cell lines. [file FEB4-9999-0-s001.pdf]

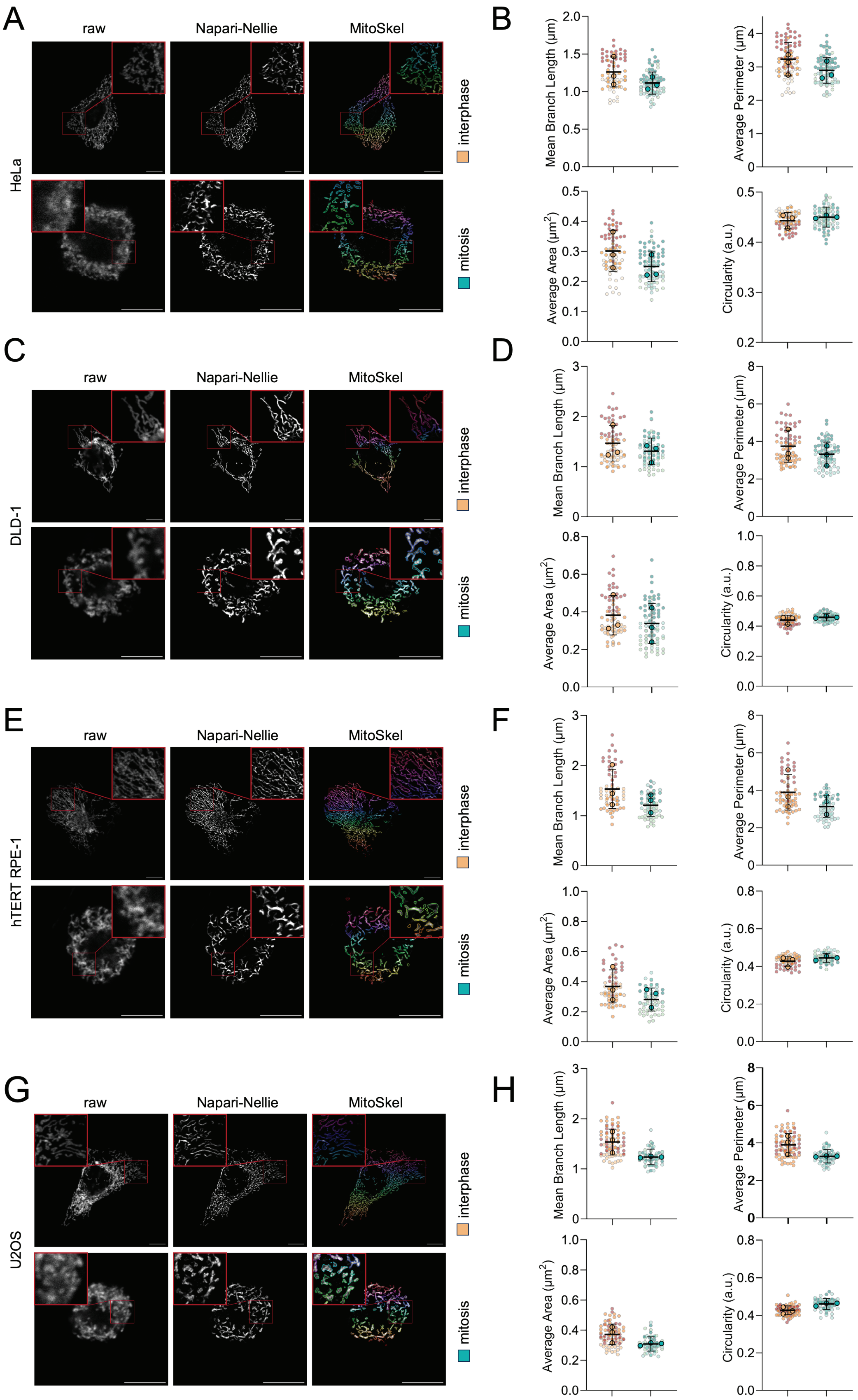
A
raw
Napari-Nellie
MitoSkel
HeLa
interphase
mitosis
B
Mean Branch Length (µm)
Average Perimeter (µm)
Average Area (µm2)
Circularity (a.u.)
C
raw
Napari-Nellie
MitoSkel
DLD-1
interphase
mitosis
D
Mean Branch Length (µm)
Average Perimeter (µm)
Average Area (µm2)
Circularity (a.u.)
E
raw
Napari-Nellie
MitoSkel
hTERT RPE-1
interphase
mitosis
F
Mean Branch Length (µm)
Average Perimeter (µm)
Average Area (µm2)
Circularity (a.u.)
G
raw
Napari-Nellie
MitoSkel
U2OS
interphase
mitosis
H
Mean Branch Length (µm)
Average Perimeter (µm)
Average Area (µm2)
Circularity (a.u.)

Figure S1. Application of RoundMi to multiple cell lines. **A**. Representative confocal images of fixed HeLa cells in interphase and mitosis. Images were pre-processed using Napari-Nellie and segmented using MitoSkel. Images of mitotic cells were cropped in ImageJ/Fiji to provide a slightly zoomed-in view. Scale bar: 10 µm. **B**. Quantification of mitochondrial morphology parameters in HeLa cells during interphase and mitosis following RoundMi workflow. **C**. Representative confocal images of DLD-1 cells in interphase and mitosis. **D**. Quantification of mitochondrial morphology parameters in DLD-1 cells as in (**B**). **E**. Representative confocal images of hTERT RPE-1 cells in interphase and mitosis. (**F**) Quantification of mitochondrial morphology parameters in hTERT RPE-1 cells as in (**B**). **G**. Representative confocal images of U2OS cells in interphase and mitosis. **H**. Quantification of mitochondrial morphology parameters in U2OS cells as in (**B**). TOMM20 is used to visualize mitochondria. Data represent $n = 3$ independent experiments, with approximately 25 cells analyzed per replica (~ 75 cells) per condition. Transparent circles represent individual cells, with different colors indicating independent replicates. Filled circles indicate the mean of each replicate, the black line indicates the mean of all cells and error bars represent standard deviation. Color coding for interphase and mitosis is indicated in the figure. Pairwise comparisons between conditions were assessed using two-way ANOVA statistical analysis. *$p < 0.05$; **$p < 0.01$; ***$p < 0.001$; ****$p < 0.0001$; ns, not significant ($p > 0.05$).
